# Effect of Capsule Burst in Cigarette Filters on the Compound Composition of Mainstream Cigarette Smoke

**DOI:** 10.3390/toxics11110901

**Published:** 2023-11-03

**Authors:** Hyeon-Su Lim, Ji-Sang You, Heung-Bin Lim

**Affiliations:** Department of Industrial Plant Science and Technology, Chungbuk National University, Cheongju 28644, Republic of Korea; hu0719@chungbuk.ac.kr (H.-S.L.); r9510@naver.com (J.-S.Y.)

**Keywords:** capsule cigarette, Hoffmann list, menthofuran, pulegone, recovery rate

## Abstract

The number of cigarette capsule users is increasing; however, a comprehensive epidemiological investigation comparing the harmfulness of capsule and non-capsule cigarettes, particularly concerning the composition of flavor components and mainstream smoke, is lacking. The present study aimed to investigate the effect of capsule burst on the compound composition of mainstream smoke by quantifying the Hoffmann list and flavor (geraniol, eugenol, menthofuran, and pulegone) in mainstream smoke with and without crushing the capsules. The findings indicate that while tar values tended to increase when the capsules were burst, there was no significant change observed in the other Hoffmann list components, such as nicotine, benzo[α]pyrene, tobacco-specific nitrosamines, aromatic amines, and phenolics. However, when the capsules burst, menthofuran and pulegone, which were present in the capsule and could cause toxicity, were found in the mainstream smoke via the International Standard Organization (1.5–4.0 μg/cig and 5.13–6.00 μg/cig smoking regime) and Health Canada Intense (12.8–18.2 μg/cig and 22.77–24.67 μg/cig smoking regime). Therefore, understanding the capsule composition is important, as the toxic components of the capsules can be inhaled as smoke, posing a potential health risk.

## 1. Introduction

The demand for capsule cigarettes has steadily increased in recent years. Between 2008 and 2019, the proportion of capsule cigarette users among smokers increased from 38% to 41% [1]. Capsule cigarettes are innovative because they contain a capsule in the filter [2], which when crushed, releases flavors derived from various ingredients. Each capsule contains more than 128 compounds, including menthol or fruit extract, which offers a specific aroma, allowing smokers to select a flavor that suits their preferences [2,3,4]. However, despite their popularity, a comprehensive epidemiological investigation comparing the harmfulness of capsule and non-capsule cigarettes is lacking, particularly concerning the composition of flavor components and mainstream smoke. Based on this, we aimed to investigate the potential effects of capsule ingredients on the composition of toxic compounds in mainstream smoke.

The adverse health effects of tobacco use stem primarily from the inhalation of mainstream smoke [5]. As mainstream smoke directly enters the lungs, the ingestion of the same quantity of harmful substances through this route poses greater risks than oral consumption. Mainstream smoke contains 8000 substances, including 73 carcinogens such as polycyclic aromatic hydrocarbons (PAHs), aldehydes, and tobacco-specific nitrosamines (TSNAs) [6,7]. Notably, regulatory authorities in the United States and Canada have identified certain substances on this extensive list as significant contributors to tobacco-related diseases [8]. Referring to the Hoffmann list, these ingredients aligned with those previously reported by Hoffmann serve as important parameters for comparing the content analysis of different cigarettes to determine their relative harmfulness.

Several studies have investigated the effects of capsules on the composition of compounds in mainstream smoke. For instance, Gordon et al. reported that the amount of tar in mainstream smoke increased upon capsule bursting [9]. Furthermore, Dolka et al. identified a correlation between the menthol content and the increased levels of volatile organic compounds (VOCs) in mainstream smoke [10]. However, these studies often overlooked the effect of filter circumference and length on compound amounts and predominantly focused on king-size cigarettes [11]. Because the length and circumference of the filter can affect the composition of mainstream smoke, it is imperative to explore other cigarette sizes. Additionally, although an increase in tar content has been attributed to the transport of particulate components from capsules, there is a lack of research confirming its occurrence.

To address these questions, in this study, we aimed to compare the contents of the Hoffmann list with and without capsule crushing by analyzing mainstream smoke from both king-size and super-slim cigarettes. The analysis adhered to the International Standard Organization (ISO) and Health Canada Intense (HCI) guidelines for the systematic analysis of the Hoffmann list. Furthermore, potentially hazardous compounds reported to be present in the capsules were selected, and quantitative analysis was performed using gas chromatography–mass spectrometry (GC–MS) on the capsules and mainstream smoke samples. By calculating the delivery rate based on the obtained content values, a comparative analysis was conducted across the different smoking methods (ISO and HCI) and cigarette sizes.

## 2. Materials and Methods

### 2.1. Materials

Capsule cigarette samples were selected from the four cigarettes that were the best-selling cigarettes in Korea among regular and super-slim cigarettes [12]. Samples A and C and B and D are of the same brand of cigarettes, respectively. These cigarettes were purchased from a Korean market and smoked after conditioning for 48 h at constant temperature and humidity of 22 ± 1 °C and 60 ± 3%, respectively. As a reference cigarette, Kentucky 3R4F cigarettes provided by the Ministry of Food and Drug Safety in Korea were used and conditioned at constant temperature and humidity under the same conditions as before smoking. Each capsule of cigarettes was obtained by cutting the cigarette filter containing the capsule. Standards of geraniol, eugenol, menthofuran, and pulegone were purchased from Merck (Darmstadt, Germany). The physical data of cigarettes and capsules are summarized in Table 1.

### 2.2. Analytical Methods

#### 2.2.1. Hoffmann List

The cigarettes were smoked using a smoking machine RM20H (Heinr Borgwaldt, Germany) until the desired butt length (filter length + 3 mm) was obtained according to the ISO 3308 smoking regime [13] and the HCI T-115 smoking regime [14]. The ISO smoking regimen included a puff volume of 35 mL, puff duration of 2 s, and one puff per min. The HCI smoking regime is an increased smoking regime, in which the puff volume is increased to 55 mL, the frequency of puffs per min is increased to 2, and the ventilation holes are blocked. Cigarette-containing capsules were divided into two types—when the capsule burst (CB) and when the capsule did not burst (NCB)—and mainstream smoke was collected from each group. The capsule was burst using a capsule crushing device to ensure that the content in the capsule was consistently released, and the mainstream smoke from the CB group was collected 5 ± 1 min after crushing the capsule.

The Hoffmann list compounds were analyzed using a previously validated method provided by the ISO. For compounds without the analysis method provided by the ISO, the method recommended by HCI was used. The compounds to be analyzed and their analysis methods are summarized in Table 2. Tar content was calculated by subtracting the water and nicotine values from the total particulate matter (TPM) values. Water and nicotine were analyzed using a GC–thermal conductivity detector (GC–TCD) and a GC–flame ionization detector (GC-FID) in 2-propanol extracts of TPM according to ISO 10315 and ISO 10362, respectively [15,16]. Carbon monoxide was measured using non-dispersive infrared (NDIR) according to ISO 8454 [17]. Ammonia was extracted by combining a Cambridge filter with two impingers, each containing 15 mL of 0.01 M sulfuric acid, and it was measured using ion chromatography (IC) according to ISO 23919 [18]. Aromatic amine groups, including 1 and 2-aminonaphthalene and 3 and 4–aminobiphenyl, were measured using GC–MS after extraction from TPM with 5% HCl and purification by liquid–liquid extraction and solid-phase extraction (SPE) according to T-102 [19]. Benzo[α]pyrene was extracted from the TPM using MeOH, purified by SPE, concentrated using a nitrogen evaporator, reconstituted with 1 mL cyclohexane, and measured using GC–MS according to ISO 22364 [20]. VOCs and semi-VOCs were extracted by combining a Cambridge filter with two cryogenic impingers, each containing 10 mL of MeOH, and analyzed using GC–MS according to T-116 and T-112, respectively [21,22]. Hydrogen cyanide was extracted by combining a Cambridge filter with an impinger containing 40 mL 0.1 NaOH and analyzed according to T-107 [23]. Carbonyls containing acetaldehyde, acetone, acrolein, 2-butanone, butyraldehyde, crotonaldehyde, and formaldehyde were extracted from only two impingers, each containing 35 mL of the 2,4-dinitrophenylhydrazine solution to derivatize carbonyls without filtering the mainstream smoke with a Cambridge filter, and they were analyzed using a high-performance liquid chromatography (HPLC)-photodiode array (Photodiode Array) according to ISO 23922 [24]. Phenolics containing catechol, m-cresol, o-cresol, p-cresol, phenol, and resorcinol were extracted from Cambridge filter with 1% acetic acid in deionized water (DIW) and analyzed using HPLC-fluorescence detection (Fluorescence Detector) according to ISO-23905 [25].

NNN(N-nitrosonornicotine), NNK (4-(methylnitrosamino)-1-(3-pyridyl)-1-butanone), NAT (N-nitrosoanatabine), and NAB (N-nitrosoanabasine) were extracted from the TPM using 100mM ammonium acetate in DIW and analyzed by LC-MS/MS according to ISO 19290 [26]. Heavy metals, including arsenic, cadmium, chromium, nickel, lead, selenium, and mercury, were extracted from the TPM collected from the electrostatic smoke traps, but not from the Cambridge filter. After dissolving the TPM in a smoked trap with methanol, the dissolved solution was transferred to a Teflon microwave digestion vessel and evaporated on a hot plate. The residue from the vessel was extracted by microwave digestion with hydrochloric acid, diluted with water, and analyzed using inductively coupled plasma mass spectrometry (ICP-MS) according to T-108 for mercury and T-109 for other metals [27,28]. All analyses were performed in triplicate, and it was confirmed that the content of each component of 3R4F, the reference cigarette, was within the certification value provided.

#### 2.2.2. Flavor Compound in Capsule (FCC)

After placing the capsule in a 15 mL conical tube containing 5 mL MeOH, the capsule was burst and shaken for 5 min. Capsule extracts were analyzed by GC–MS (7890/5977B, Agilent, Santa Clara, CA, USA) using the single ion monitoring (SIM) mode with eugenol, geraniol, menthofuran, and pulegone standards. The sample was injected into HP-5MS Ultra Inert (60 m × 250 μm I.D. × 0.25 μm) column under inlet temperature 280 °C and split mode (5:1) conditions using an autosampler. The carrier gas was He gas at a flow rate of 0.7 mL/min, and the oven temperature was set as follows: from 40 °C (held for 2 min) to 300 °C (held for 10 min) in increments of 5 °C/min.

### 2.3. Statistical Analysis

All results of the quantification and toxicological assays were expressed as mean ± standard deviation (SD) and performed in triplicate. Duncan’s multiple comparison tests were performed for pairwise comparisons. The statistical analyses were performed using GraphPad Prism 9.5.1 (GraphPad Software Inc, San Diego, CA, USA). Statistical significance was set at *p* < 0.05.

## 3. Results

### 3.1. Hoffmann List in the Mainstream Smoke

A Hoffmann list analysis of mainstream smoke was performed when the capsule did and did not burst. Table 3 and Table 4 show the yield of compounds contained in one cigarette when smoked by the ISO smoking regime and the HCI smoking regime, respectively. The analytical method was validated by confirming that the 3R4F yield for each compound was within the certified range. Calibration curves for all compounds showed excellent linearity (*r*^2^ = 0.99).

In the Hoffmann list, only tar content showed an increasing trend for all cigarettes when the capsules burst. This trend was more pronounced in the HCI smoking regime than in the ISO smoking regime. However, crushing the capsule tended to decrease only the VOC levels in all tested cigarettes; the decrease in VOC levels between cigarettes with the ISO regimen and those with the HCI regimen was similar. These results are consistent with previous findings [10]. However, notably, the yield of carbonyls, except crotonaldehyde, in cigarettes increased remarkably when the capsular burst was performed under the HCI regime compared to when the capsule burst was performed under the ISO regime. In contrast, no significant differences or increasing/decreasing trends were observed for other compounds, such as nicotine, benzo[α]pyrene, ammonia, aromatic amines, hydrogen cyanide, phenolics, and heavy metals.

### 3.2. Flavor Compound in Capsule (FCC) in Capsules and Mainstream

Prior to the FCC analysis of mainstream smoke, the content of the target compounds in each cigarette capsule sample was quantified. Samples A and C, which were manufactured at PMI, and samples B and D, which were manufactured at KT&G, had comparable amounts of the target compounds. Menthofuran contained 43–46 μg/cig and 21–29 μg/cig, respectively, and pulegone contained 19–21 μg/cig and 18–21 μg/cig in each cigarette, but no detectable eugenol and geraniol in either cigarette brand (Table 5). As expected, eugenol and geraniol were not detected in the mainstream smoke of any cigarettes.

**Figure 1:**
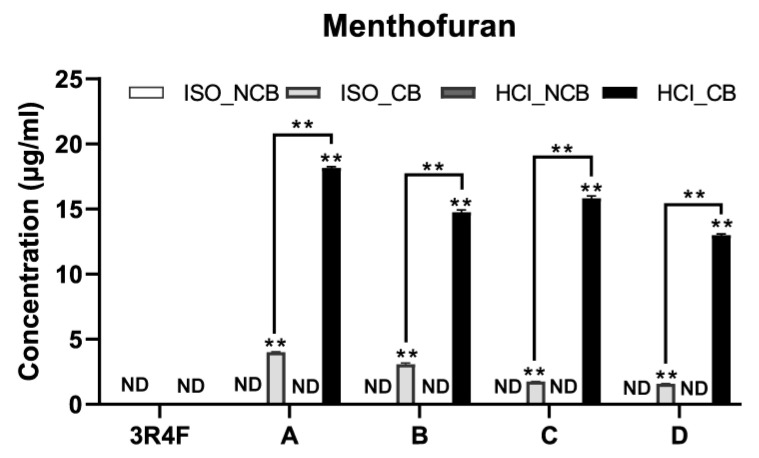
Menthofuran yield in mainstream smoke. ISO_NCB, non-capsule burst smoked by ISO smoking regime; ISO_CB, capsule burst smoked by ISO smoking regime; HCI_NCB, non-capsule burst smoked by HCI smoking regime; HCI_CB capsule burst smoked by HCI smoking regime; ND, not detectable. ** Significant difference from NCB (*p* < 0.05).

**Figure 2:**
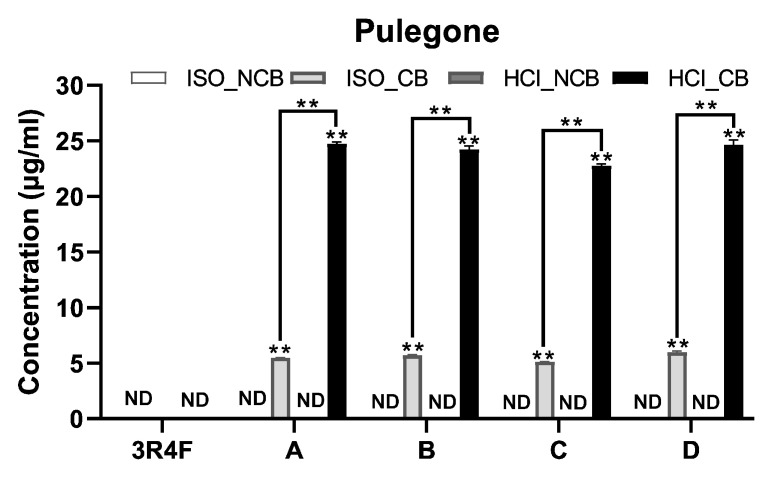
Pulegone yield in mainstream smoke. ISO_NCB, non-capsule burst smoked by ISO smoking regime; ISO_CB, capsule burst smoked by ISO smoking regime; HCI_NCB, non-capsule burst smoked by HCI smoking regime; HCI_CB capsule burst smoked by HCI smoking regime; ND, not detectable. ** Significant difference from NCB (*p* < 0.05).

However, menthofuran and pulegone were detected only when the capsules burst, and their contents significantly increased (Figure 1 and Figure 2). When smoked by the ISO smoking regime, menthofuran was quantified to be approximately 3.00–4.00 μg/cig and appeared in high-tar cigarettes A and B, and 1.57–1.75 μg/cig appeared in low-tar cigarettes C and D. When smoked by the HCI smoking regime, A and B were 14.75–18.15 μg/cig, whereas C and D were 12.98–15.82 μg/cig. Menthofuran was found to be higher in low-tar cigarettes than in high-tar cigarettes in both the ISO and HCI smoking regimes. A considerable increase was observed when smoking in the HCI smoking regime, but not in the ISO smoking regime. However, pulegone was observed at 5.13–6.00 ug/cig regardless of the amount of tar in the ISO smoking regime and 22.77–24.80 μg/cig regardless of the amount of tar in the HC smoking regime. A significant increase in menthofuran was observed when smoking in the HCI smoking regime compared to when smoking in the ISO smoking regime.

In addition, using these data, the recovery rate from the capsule to mainstream smoke for each compound was calculated and summarized in Table 6. Interestingly, different recovery rates were observed for the cigarette size, smoking method, and amount of tar and substances. Under the ISO smoking regime, menthofuran showed recovery rates of 13.79% and 14.03% for groups A and B, respectively, and 4.96% and 5.73% for groups C and D, respectively. Pulegone also showed recovery rates of 29.25% and 30.75% for A and B, respectively, and 13.29% and 14.11% for C and D, respectively In the HCI smoking regimen, menthofuran showed a higher recovery rate in Group D (62.89%) than in Group A (50.91%). Moreover, pulegone showed recovery rates of 100 or higher. The recovery rate increased significantly in the HCI smoking regime compared with that in the ISO regime. The recovery rate of pulegone is higher than that of menthofuran. Moreover, in the HCI smoking regime, menthofurans tended to have a higher overall recovery rate from super-slim-size cigarettes than from king-size cigarettes.

## 4. Discussion

The findings of this study have important implications for understanding the composition of mainstream smoke when the capsules in the cigarette filters break down. It was evident from the results that the constituents of the capsule were delivered to the mainstream smoke, thereby directly affecting its composition. In this study, we found that crushing the capsule did not have a significant effect on the Hoffmann list except for tar. Furthermore, the changes in tar content and VOC content are consistent with those of previous studies [10], regardless of cigarette size. However, an unexpected change was observed in the carbonyl content when the capsule burst under the HCI, rather than ISO smoking regime was used. Carbonyl compounds are primarily produced via combustion and thermal decomposition [29]. In contrast to the ISO smoking regime, HCI smoking involves higher heat in the tobacco rods [30] and blocked ventilation holes. Based on these facts, we hypothesized that the capsule components might have migrated into the tobacco rod and converted to carbonyl compounds at higher temperatures. However, further experiments are needed to validate this hypothesis and understand the precise mechanism underlying this conversion process.

The most crucial finding of this study was the identification of specific components, menthofuran and pulegone, that migrated into the mainstream smoke when the capsule burst. These compounds were detected only when a cigarette was smoked after bursting the capsule and when the capsule removed from the filter was burst in a conical tube containing MeOH. They were not detected when the capsule did not burst. The capsule is composed of a circular membrane made of agar containing various scent ingredients, which confirmed that when the capsule was burst, the menthofuran and pulegone contained within the capsule were released and delivered with the mainstream smoke during smoking. This explains the increase in the tar content of cigarettes when the capsule is burst, as the ingredients in the capsule are delivered into mainstream smoke. Although toxicity evaluation experiments were not conducted in this study, menthofuran and pulegone, which have been reported as toxic substances [31,32], are delivered into mainstream smoke when the capsule is burst and may have harmful effects. Therefore, to assess the toxicity of capsule cigarettes, it is essential to understand which capsule components are transported in mainstream smoke.

In this study, we also found that the rate at which capsule components are delivered to mainstream smoke (recovery) is affected by the tar value of the cigarette, the smoking method. Both king-size cigarettes (A and C samples) and super-slim size cigarettes (B and D samples) exhibited a high recovery rate of high FCC in cigarettes with high original tar value. This appears to be related to ventilation. Typically, the tar value is adjusted by adjusting the ventilation hole of the tobacco as well as the tobacco leaf used. Low-tar cigarettes have a high ventilation rate, which reduces FCC through ventilation. Additionally, ventilation explains why recovery is higher when cigarettes are burned in the HCI smoking regime than in the ISO smoking regime. As HCI blocks the ventilation hole and burns the cigarette, it appears to show a high recovery rate in the ISO smoking regime without leaks. These patterns were consistent for both king-size and super-slim size cigarettes. However, the effect of cigarette size on FCC recovery rate is not confirmed because in order to investigate this, cigarettes must be manufactured directly with cigarettes differing in size alone and consistent in factors such as tobacco leaves, capsules, and cigarette paper. This is not feasible due to the lack of technology for such cigarette production. Further research is needed in this regard.

Although this study focused on menthofuran and pulegone, different types of cigarettes contain various ingredients in their capsules. Therefore, a comprehensive evaluation of the ingredients used in the capsules and their associated toxicities should be conducted using a wide range of cigarettes. We confirmed that certain substances with individual toxicity can be found in mainstream smoke. However, whether the toxicity increases when the capsule is crushed was not confirmed. Therefore, future research should focus on the assessment of the toxic effects of these substances to provide a more comprehensive understanding of their potential risks.

## 5. Conclusions

To conclude, this study indicated that the constituents of capsules in cigarette filters are delivered to mainstream smoke, significantly affecting its composition. The identification of menthofuran and pulegone as components transported into smoke highlights the potential health risks associated with capsule cigarette use. Furthermore, they exhibit different recovery rates depending on factors such as tar content, cigarette size, and composition. These findings contribute to our understanding of the potential health risks associated with cigarette capsules and highlight the need for further research and regulations in this area.

## Figures and Tables

**Table 1:** Physical data of sample cigarettes and their capsules.

Sample	3R4F	A	B	C	D
Manufacturer	Kentucky University	PMI	KT&G	PMI	KT&G
Tar Level (mg/cig)	9.4 ± 1.38	6	4	1	1
Cigarette length (mm)	84 ± 0.5	83 ± 0.5	100 ± 0.5	83 ± 0.5	100 ± 0.5
Filter length (mm)	27 ± 0.5	25 ± 0.5	30 ± 0.5	25 ± 0.5	30 ± 0.5
Circumference (mm)	25.13 ± 3.14	25.13 ± 3.14	17.28± 3.14	25.13 ± 3.14	17.28 ± 3.14
Capsule weight (mg)	-	20.8 ± 0.1	10.7 ± 0.2	21.2 ± 0.1	10.9 ± 0.2
Size	KS	KS	SS	KS	SS

PMI: Philip Morris Incorporation, KT&G: Korea Tomorrow & Global Corporation, KS: King size, SS: Super-slim size.

**Table 2:** Method used for Hoffmann list quantification of cigarette mainstream smoke.

Group	Compound	Analyzing Method
-	Nicotine	ISO 10315
	Tar	ISO 10362
	CO	ISO 8454
Amine	Ammonia	ISO 23919
Aromatic amine	1 and 2-aminonaphthalene, 3 and 4—aminobiphenyl	T-102
Poly hydrocarbon (PAH)	benzo[α]pyrene	ISO 22364
Volatile Organic Compound (VOC)	acrylonitrile, benzene, 1,3-butadiene, isoprene, toluene	T-116
Semi-Volatile Organic Compound (Semi-VOC)	pyridine, quinoline, styrene	T-112
Cyanic compound	Hydrogen cyanide	T-107
Carbonyls	acetaldehyde, acetone, acrolein, 2-butanone, butyraldehyde, crotonaldehyde, formaldehyde, propionaldehyde	ISO 23922
Phenolics	catechol, m-cresol, o-cresol, p-cresol, hydroquinone, phenol, resorcinol	ISO 23905
Tobacco-specific nitrosamines	N-nitrosonornicotine (NNN), 4-(methylnitrosamino)-1-(3-pyridyl)-1-butanone (NNK), N-nitrosoanatabine (NAT), N-nitrosoanabasine (NAB)	ISO 19290
Heavy metal	Arsenic (As), cadmium (Cd), chromium (Cr), nickel (Ni), lead (Pb), selenium (Se)	T-109
	Mercury (Hg)	T-108

**Table 3:** Yield of Hoffmann list smoked by ISO smoking regime.

Analytes	Unit	3R4F	A		B		C		D	
			CB	NCB	CB	NCB	CB	NCB	CB	NCB
Tar	mg/cig	8.43 ± 0.25	5.91 ± 0.49	5.20 ± 0.11	4.53 ± 0.13	3.72 ± 0.20	1.21 ± 0.14	0.71 ± 0.14	1.31 ± 0.06	0.8 ± 0.21
Nicotine	mg/cig	0.70 ± 0.01	0.44 ± 0.02	0.45 ± 0.01	0.35 ± 0.01	0.35 ± 0.01	0.10 ± 0.01	0.12 ± 0.05	0.11 ± 0.01	0.11 ± 0.02
CO	mg/cig	11.51 ± 0.19	6.35 ± 0.27	6.37 ± 0.04	3.49 ± 1.47	3.41 ± 0.04	2.14 ± 0.08	2.00 ± 0.16	0.88 ± 0.09	0.86 ± 0.10
Amine										
Ammonia	µg/cig	28.03 ± 1.25	4.35 ± 0.35	4.10 ± 0.08	2.94 ± 0.14	2.55 ± 0.025	1.59 ± 0.10	1.84 ± 0.05	1.45 ± 0.08	1.57 ± 0.12
Aromatic amine										
1-aminonaphthalene	ng/cig	9.12 ± 1.71	6.01 ± 0.17	5.75 ± 0.15	1.53 ± 0.05	1.46 ± 0.03	1.50 ± 0.04	1.44 ± 0.04	0.38 ± 0.02	0.36 ± 0.02
2-aminonaphthalene	ng/cig	5.10 ± 2.79	4.26 ± 0.11	4.07 ± 0.08	0.60 ± 0.04	0.58 ± 0.05	1.07 ± 0.03	1.02 ± 0.02	0.12 ± 0.04	0.12 ± 0.04
3-aminobiphenyl	ng/cig	2.27 ± 0.15	0.96 ± 0.01	0.92 ± 0.03	0.19 ± 0.02	0.18 ± 0.02	0.24 ± 0.01	0.23 ± 0.01	N.Q	N.Q
4-aminobiphenyl	ng/cig	2.03 ± 0.15	1.40 ± 0.17	1.34 ± 0.15	0.62 ± 0.01	0.6 ± 0.01	0.35 ± 0.04	0.33 ± 0.04	N.Q	N.Q
PAH										
Benzo[α]pyrene	ng/cig	6.33 ± 0.53	7.38 ± 0.50	6.73 ± 0.47	1.71 ± 0.26	1.63 ± 0.1	3.67 ± 0.16	3.50 ± 0.17	1.37 ± 0.31	1.19 ± 0.31
Carbonyls										
Acetaldehyde	µg/cig	480.06 ± 59.17	234.09 ± 1.41	262.99 ± 0.58	126.01 ± 1.42	115.1 ± 0.38	53.41 ± 1.26	54.27 ± 0.24	30.25 ± 0.11	41.79 ± 0.09
Acetone	µg/cig	188.03 ± 24.97	24.00 ± 0.16	27.04 ± 0.23	16.50 ± 0.13	15.84 ± 0.05	9.03 ± 0.54	9.37 ± 0.02	7.93 ± 0.02	9.17 ± 0.12
Acrolein	µg/cig	45.49 ± 5.35	104.13 ± 2.49	121.43 ± 2.05	49.46 ± 1.20	51.80 ± 1.93	28.78 ± 0.99	29.26 ± 2.14	19.73 ± 1.50	26.37 ± 1.89
2-Butanone	µg/cig	59.61 ± 6.95	30.03 ± 0.16	36.11 ± 0.14	16.45 ± 0.21	18.67 ± 0.05	9.26 ± 0.37	12.48 ± 0.05	10.84 ± 0.02	12.13 ± 0.05
Butyraldehyde	µg/cig	24.96 ± 2.94	11.32 ±0.07	13.18 ± 0.05	4.68 ± 0.13	6.14 ± 0.42	2.51 ± 0.20	3.37 ± 0.05	N.Q	N.Q
Crotonaldehyde	µg/cig	11.28 ± 3.49	9.52 ± 0.01	12.44 ± 0.17	4.45 ± 0.09	5.19 ± 0.02	1.91 ± 0.39	2.21 ± 0.04	1.59 ± 0.02	1.89 ± 0.06
Formaldehyde	µg/cig	21.06 ± 5.31	21.69 ± 0.11	22.32 ± 0.05	21.3 ± 0.21	19.93 ± 0.11	18.57 ± 0.42	18.22 ± 0.02	15.70 ± 0.05	16.17 ± 0.09
Propionaldehyde	µg/cig	39.82 ± 3.61	20.01 ± 0.16	23.19 ± 0.59	10.26 ± 0.12	10.56 ± 0.09	5.83 ± 0.32	6.04 ± 0.08	4.79 ± 0.04	4.95 ± 0.15
Cyanic compound										
HCN	µg/cig	77.00 ± 1.25	55.47 ± 1.70	59.00 ± 2.29	28.25 ± 2.25	28.00 ± 1.56	4.58 ± 0.52	6.83 ± 0.38	6.08 ± 1.04	5.50 ± 0.50
Phenolics										
Catechol	µg/cig	35.85 ± 1.32	25.58 ± 0.77	25.75 ± 1.02	24.15 ± 0.55	23.99 ± 0.76	9.19 ± 0.37	9.26 ± 0.38	8.90 ± 1.39	7.95 ± 0.83
p-cresol	µg/cig	3.83 ± 0.29	0.87 ± 0.05	0.88 ± 0.05	1.31 ± 0.14	1.30 ± 0.15	0.12 ± 0.01	0.12 ± 0.01	0.13 ± 0.02	0.13 ± 0.02
m-cresol	µg/cig	1.36 ± 0.05	0.91 ± 0.04	0.91 ± 0.04	1.05 ± 0.10	1.04 ± 0.11	0.10 ± 0.01	0.10 ± 0.01	0.11 ± 0.01	0.11 ± 0.01
o-cresol	µg/cig	2.20 ± 0.57	2.28 ± 0.08	2.29 ± 0.10	2.86 ± 0.29	2.84 ± 0.31	0.26 ± 0.01	0.27 ± 0.01	0.29 ± 0.03	0.29 ± 0.03
Hydroquinone	µg/cig	37.12 ± 1.79	23.92 ± 1.22	24.07 ± 1.18	23.48 ± 0.08	23.33 ± 0.34	8.78 ± 0.12	8.84 ± 0.19	8.41 ± 1.05	8.12 ± 0.37
Phenol	µg/cig	7.88 ± 0.55	2.73 ± 0.11	2.75 ± 0.14	5.03 ± 0.78	5.00 ± 0.79	0.31 ± 0.01	0.31 ± 0.01	0.74 ± 0.17	0.74 ± 0.16
Resorcinol	µg/cig	0.69 ± 0.19	0.41 ± 0.04	0.41 ± 0.04	0.44 ± 0.01	0.44 ± 0.02	0.24 ± 0.01	0.41 ± 0.04	0.16 ± 0.03	0.16 ± 0.03
Semi-Volatile organic compounds										
Pyridine	µg/cig	25.47 ± 1.50	2.31 ± 0.12	2.81 ± 0.05	1.43 ± 0.01	1.92 ± 0.03	2.00 ± 1.62	1.31 ± 0.03	0.72 ± 0.02	0.50 ± 0.03
Quinoline	µg/cig	0.37 ± 0.07	0.14 ± 0.01	0.10 ± 0.02	0.08 ± 0.01	0.08 ± 0.01	N.Q	N.Q	N.Q	N.Q
Styrene	µg/cig	13.94 ±	0.96 ± 0.02	1.65 ± 0.03	0.53 ± 0.01	0.92 ± 0.01	N.Q	N.Q	N.Q	N.Q
Tobacco-specific nitrosamines										
NNN	ng/cig	107.12 ± 1.37	40.93 ± 3.26	40.53 ± 2.60	15.32 ± 3.23	12.78 ± 3.89	10.65 ± 1.13	10.63 ± 1.02	4.84 ± 3.58	1.55 ± 0.79
NNK	ng/cig	90.56 ± 2.03	18.14 ± 1.53	18.31 ± 0.62	6.52 ± 0.62	4.07 ± 3.49	2.97 ± 0.53	2.95 ± 0.60	N.Q	N.Q
NAT	ng/cig	119.08 ± 3.40	56.59 ± 3.19	56.43 ± 0.62	21.43 ± 4.52	17.56 ± 4.27	14.87 ± 1.17	14.56 ± 0.64	5.67 ± 0.20	3.12 ± 1.05
NAB	ng/cig	13.37 ± 0.88	7.96 ± 0.72	7.27 ± 1.07	4.85 ± 1.46	4.61 ± 1.16	3.02 ± 0.78	2.83 ± 0.85	3.31 ± 0.12	3.35 ± 0.52
Volatile organic compounds										
Acrylonitrile	µg/cig	9.70 ± 0.60	4.79 ± 0.04	5.46 ± 0.06	2.62 ± 0.05	3.03 ± 0.06	1.03 ± 0.04	1.50 ± 0.04	0.65 ± 0.01	0.89 ± 0.02
Benzene	µg/cig	37.34 ± 1.96	20.85 ± 0.03	23.3 ± 0.04	4.04 ± 0.01	9.22 ± 0.01	0.53 ± 0.05	4.35 ± 0.01	N.Q	N.Q
1,3-butadiene	µg/cig	25.77 ± 2.86	12.18 ± 0.12	13.22 ± 0.03	6.71 ± 1.44	6.87 ± 0.43	2.25 ± 0.36	3.01 ± 0.65	0.93 ± 0.28	2.37 ± 0.25
Isoprene	µg/cig	293.52 ± 13.02	142.33 ± 0.82	162.69 ± 1.94	74.03 ± 1.74	86.70 ± 1.75	25.14 ± 1.29	38.51 ± 1.53	11.54 ± 0.45	19.17 ± 0.44
Toluene	µg/cig	70.28 ± 5.46	33.11 ± 1.27	40.78 ± 0.17	5.91 ± 0.81	16.15 ± 2.10	2.62 ± 0.42	4.32 ± 0.18	N.Q	N.Q
Heavy metal										
Arsenic	ng/cig	1.91 ± 0.08	N.Q	N.Q	N.Q	N.Q	N.Q	N.Q	N.Q	N.Q
Cadmium	ng/cig	32.52 ± 2.09	25.16 ± 0.27	22.75 ± 0.08	3.96 ± 0.05	3.85 ± 0.04	5.25 ± 0.07	2.64 ± 0.07	1.19 ± 0.04	0.34 ± 0.02
Chromium	ng/cig	0.19 ± 0.14	N.Q	N.Q	N.Q	N.Q	N.Q	N.Q	N.Q	N.Q
Mercury	ng/cig	N.Q	N.Q	N.Q	N.Q	N.Q	N.Q	N.Q	N.Q	N.Q
Nickel	ng/cig	0.96 ± 0.08	N.Q	N.Q	N.Q	N.Q	N.Q	N.Q	N.Q	N.Q
Lead	ng/cig	8.84 ± 0.83	7.84 ± 0.08	7.01 ± 0.06	3.09 ± 0.04	2.14 ± 0.18	N.Q	N.Q	N.Q	N.Q
Selenium	ng/cig	0.56 ± 0.25	N.Q	N.Q	N.Q	N.Q	N.Q	N.Q	N.Q	N.Q

N.Q: not quantifiable.

**Table 4:** Yield of Hoffmann list smoked by HCI smoking regime.

Analytes	Unit	HCI_3R4F	HCI_A		HCI_B		HCI_C		HCI_D	
			CB	NCB	CB	NCB	CB	NCB	CB	NCB
Tar	mg/cig	27.93 ± 0.77	37.30 ± 1.2	33.58 ± 0.99	31.13 ± 0.94	28.49 ± 1.67	28.52 ± 0.47	25.97 ± 0.73	29.06 ± 1.66	27.2 ± 0.48
Nicotine	mg/cig	1.93 ± 0.07	1.51 ± 0.08	1.43 ± 0.17	1.39 ± 0.05	1.45 ± 0.07	1.14 ± 0.01	1.03 ± 0.06	1.28 ± 0.04	1.32 ± 0.07
CO	mg/cig	37.01 ± 0.96	27.58 ± 1.06	27.35 ± 0.4	19.01 ± 0.39	18.95 ± 0.52	27.15 ± 1.27	26.9 ± 0.19	18.65 ± 0.39	18.94 ± 0.39
Amine										
Ammonia	µg/cig	26.90 ± 0.34	24.99 ± 0.17	26.17 ± 0.22	24.08 ± 17.9	25.44 ± 0.13	26.37 ± 0.20	25.17 ± 0.11	25.63 ± 0.2	24.79 ± 0.23
Aromatic amine										
1-aminonaphthalene	ng/cig	23.32 ± 3.67	15.88 ± 0.27	15.19 ± 0.28	2.3 ± 0.13	2.2 ± 0.16	3.97 ± 0.07	3.80 ± 0.07	0.83 ± 0.04	0.79 ± 0.05
2-aminonaphthalene	ng/cig	15.06 ± 7.36	6.76 ± 0.23	6.47 ± 0.18	3.87 ± 0.30	3.71 ± 0.36	1.69 ± 0.06	1.62 ± 0.04	0.46 ± 0.10	0.44 ± 0.09
3-aminobiphenyl	ng/cig	5.94 ± 1.76	1.09 ± 0.31	1.04 ± 0.29	1.55 ± 0.19	1.48 ± 0.20	0.27 ± 0.08	0.26 ± 0.07	0.37 ± 0.01	0.36 ± 0.01
4-aminobiphenyl	ng/cig	4.08 ± 1.40	2.17 ± 0.35	2.08 ± 0.37	1.34 ± 0.05	1.28 ± 0.06	0.54 ± 0.08	0.52 ± 0.09	0.09 ± 0.01	0.08 ± 0.01
PAH										
Benzo[α]pyrene	ng/cig	13.83 ± 2.51	18.49 ± 0.63	18.17 ± 0.41	8.74 ± 0.29	9.27 ± 0.17	12.03 ± 0.38	12.39 ± 0.33	12.09 ± 0.5	12.08 ± 0.01
Carbonyls										
Acetaldehyde	µg/cig	1300.53 ± 33.51	1345.17 ± 6.01	1207.79 ± 3.14	921.00 ± 44.39	980.03 ± 3.65	1532.5 ± 43.01	1187.29 ± 9.23	865.93 ± 5.46	372.34 ± 3.19
Acetone	µg/cig	529.30 ± 55.04	161.15 ± 0.06	153.36 ± 0.41	117.31 ± 5.22	129.38 ± 0.53	168.18 ± 11.16	141.40 ± 1.22	116.55 ± 0.72	51.63 ± 0.86
Acrolein	µg/cig	162.87 ± 29.96	507.27 ± 3.48	468.91 ± 2.63	320.34 ± 18.12	341.28 ± 2.51	568.84 ± 16.14	451.65 ± 5.78	294.53 ± 2.44	145.05 ± 3.86
2-Butanone	µg/cig	163.29 ± 17.10	145.69 ± 0.92	135.80 ± 0.38	97.24 ± 4.52	100.42 ± 3.86	171.79 ± 0.96	131.66 ± 1.29	91.44 ± 0.75	51.07 ± 2.10
Butyraldehyde	µg/cig	65.13 ± 8.19	55.88 ± 0.28	52.82 ± 0.22	39.11 ± 1.75	40.83 ± 1.08	69.15 ± 5.89	51.25 ± 0.53	35.23 ± 0.22	18.00 ± 0.35
Crotonaldehyde	µg/cig	36.92 ± 4.60	22.98 ± 14.45	13.1 ± 12.91	9.98 ± 8.64	14.23 ± 10.41	31.09 ± 1.54	24.85 ± 0.18	16.16 ± 9.38	17.24 ± 6.76
Formaldehyde	µg/cig	95.54 ± 7.51	127.6 ± 0.35	107.42 ± 0.31	121.98 ± 3.59	147.15 ± 1.79	116.58 ± 5.61	84.67 ± 0.39	123.7 ± 0.57	48.07 ± 0.18
Propionaldehyde	µg/cig	109.00 ± 24.13	112.18 ± 0.66	101.88 ± 0.27	86.51 ± 7.64	95.08 ± 1.19	104.91 ± 5.01	94.44 ± 0.84	82.75 ± 0.74	34.56 ± 2.5
Cyanic compound										
HCN	µg/cig	355.60 ± 4.24	379.58 ± 10.65	361.67 ± 0.95	263.54 ± 27.17	281.04 ± 5.91	335.63 ± 1577	304.17 ± 10.18	195.42 ± 11.01	211.04 ± 1.91
Phenolics										
Catechol	µg/cig	96.70 ± 0.25	44.41 ± 1.31	44.7 ± 0.69	85.26 ± 0.82	85.85 ± 5.05	57.88 ± 1.39	58.25 ± 0.51	76.64 ± 3.26	77.12 ± 2.24
p-cresol	µg/cig	8.72 ± 0.39	2.74 ± 0.21	2.76 ± 0.17	3.74 ± 0.17	3.76 ± 0.12	2.12 ± 0.23	2.14 ± 0.26	3.66 ± 0.13	3.69 ± 0.09
m-cresol	µg/cig	3.20 ± 0.13	2.33 ± 0.16	2.34 ± 0.12	3.00 ± 0.08	3.02 ± 0.04	1.23 ± 0.01	1.23 ± 0.02	76.64 ± 3.26	77.12 ± 2.24
o-cresol	µg/cig	4.22 ± 0.07	6.43 ± 0.47	6.46 ± 0.38	8.93 ± 0.41	8.98 ± 0.28	3.55 ± 0.41	3.58 ± 0.44	8.75 ± 0.32	8.8 ± 0.20
Hydroquinone	µg/cig	99.30 ± 3.19	64.13 ± 2.72	64.53 ± 1.74	85.00 ± 3.77	85.60 ± 4.97	61.75 ± 1.49	62.15 ± 0.79	74.18 ± 4.34	74.64 ± 3.56
Phenol	µg/cig	12.15 ± 0.30	8.04 ± 0.71	8.09 ± 0.60	14.72 ± 0.82	14.81 ± 0.61	6.53 ± 0.25	6.57 ± 0.24	13.83 ± 1.08	13.91 ± 0.90
Resorcinol	µg/cig	1.63 ± 0.19	1.02 ± 0.07	1.03 ± 0.06	1.68 ± 0.07	1.69 ± 0.09	1.18 ± 0.02	1.19 ± 0.04	1.98 ± 0.10	1.99 ± 0.09
Semi-Volatile organic compounds										
Pyridine	µg/cig	25.47 ± 1.50	21.59 ± 1.77	24.51 ± 1.90	15.75 ± 1.71	14.83 ± 0.46	13.81 ± 0.57	15.55 ± 0.04	14.17 ± 0.30	14.76 ± 1.13
Quinoline	µg/cig	0.37 ± 0.07	0.58 ± 0.06	0.98 ± 0.03	0.96 ± 0.2	1.31 ± 0.43	0.19 ± 0.02	0.46 ± 0.06	0.64 ± 0.10	1.19 ± 0.39
Styrene	µg/cig	13.94 ± 1.72	9.16 ± 0.10	10.03 ± 0.12	5.69 ± 0.03	7.01 ± 0.15	6.78 ± 0.26	8.71 ± 0.09	5.16 ± 0.11	5.89 ± 0.08
Tobacco-specific nitrosamines										
NNN	ng/cig	267.10 ± 24.41	154.4 ± 10.65	168.34 ± 18.96	62.2 ± 3.61	68.53 ± 4.59	134.74 ± 15.78	106.08 ± 19.07	68.53 ± 4.59	93.03 ± 5.91
NNK	ng/cig	243.05 ± 17.16	53.22 ± 10.22	50.8 ± 1.7	47.6 ± 3.42	37.71 ± 1.82	53.83 ± 3.29	34.92 ± 4.50	28.95 ± 1.54	28.67 ± 4.80
NAT	ng/cig	320.14 ± 19.89	207.2 ± 31.94	204.56 ± 29.48	110.99 ± 4.67	95.21 ± 4.26	160.74 ± 12.06	153.34 ± 11.40	79.79 ± 4.04	81.71 ± 7.00
NAB	ng/cig	32.73 ± 5.66	30.27 ± 1.35	29.21 ± 2.8	16.10 ± 5	14.36 ± 6.24	23.91 ± 1.31	18.68 ± 2.77	13.13 ± 5.05	13.25 ± 9.51
Volatile organic compounds										
Acrylonitrile	µg/cig	33.96 ± 3.24	21.06 ± 0.22	22.59 ± 0.26	16.92 ± 0.33	18.31 ± 0.22	19.57 ± 1.00	21.59 ± 0.04	14.17 ± 0.13	16.54 ± 0.36
Benzene	µg/cig	105.43 ± 0.38	81.5 ± 0.06	84.01 ± 0.15	46.64 ± 0.05	51.49 ± 0.11	74.57 ± 1.05	77.8 ± 0.13	40.33 ± 0.02	44.64 ± 0.08
1,3-butadiene	µg/cig	60.15 ± 9.60	56.72 ± 0.6	57.15 ± 5.24	40.64 ± 0.73	43.19 ± 0.30	49.24 ± 2.52	54.27 ± 1.26	36.68 ± 0.8	37.29 ± 4.68
Isoprene	µg/cig	816.75 ± 78.93	646.54 ± 7.18	691.87 ± 8.06	516.86 ± 10.24	560.92 ± 7.22	661.35 ± 21.27	665.44 ± 1.36	429.86 ± 3.93	506.08 ± 10.51
Toluene	µg/cig	209.15 ± 6.86	145.33 ± 0.65	150.75 ± 0.84	80.10 ± 0.67	86.75 ± 1.36	133.58 ± 1.81	139.06 ± 3.00	70.31 ± 1.06	76.29 ± 0.83
Heavy metal										
Arsenic	ng/cig	7.80 ± 0.79	2.94 ± 0.27	3.38 ± 0.14	N.Q	N.Q	2.89 ± 0.01	3.64 ± 0.12	N.Q	N.Q
Cadmium	ng/cig	118.21 ± 11.90	106.50 ± 0.16	160.93 ± 0.5	38.35 ± 0.23	35.01 ± 0.08	112.7 ± 0.62	110.5 ± 1.32	29.28 ± 0.18	36.49 ± 0.18
Chromium	ng/cig	1.89 ± 1.11	N.Q	N.Q	N.Q	N.Q	N.Q	N.Q	N.Q	N.Q
Mercury	ng/cig	N.Q	N.Q	N.Q	N.Q	N.Q	N.Q	N.Q	N.Q	N.Q
Nickel	ng/cig	1.06 ± 0.67	N.Q	N.Q	N.Q	N.Q	N.Q	N.Q	N.Q	N.Q
Lead	ng/cig	24.72 ± 11.11	36.29 ± 0.16	30.56 ± 0.46	15.69 ± 0.65	18.58 ± 0.22	28.54 ± 0.12	35.00 ± 0.47	11.99 ± 0.19	19.07 ± 0.15
Selenium	ng/cig	1.50 ± 1.02	N.Q	N.Q	N.Q	N.Q	N.Q	N.Q	N.Q	N.Q

N.Q: not quantifiable.

**Table 5:** Yield of FCC in capsule.

Sample	Geraniol (µg/g)	Eugenol (µg/g)	Menthofuran (µg/g)	Pulegone (µg/g)
A	N.D	N.D	44.08 ± 1.97	20.31 ± 0.82
B	N.D	N.D	21.88 ± 0.64	18.57 ± 0.06
C	N.D	N.D	45.33 ± 0.37	19.34 ± 0.10
D	N.D	N.D	27.38 ± 2.24	21.19 ± 0.77

N.D: not detectable.

**Table 6:** Recovery rate of FCC from the capsule to mainstream smoke.

Sample	Smoking Method	Geraniol (%)	Eugenol (%)	Menthofuran (%)	Pulegone (%)
A	ISO	N.D	N.D	13.79	29.25
	HCI	N.D	N.D	50.91	121.86
B	ISO	N.D	N.D	14.03	30.75
	HCI	N.D	N.D	67.41	130.49
C	ISO	N.D	N.D	4.96	13.29
	HCI	N.D	N.D	44.34	117.74
D	ISO	N.D	N.D	5.73	14.11
	HCI	N.D	N.D	62.89	116.42

N.D: not detectable.

## Data Availability

The data presented in the study are available on request from the corresponding author.
